# Life satisfaction and sustainability: a policy framework

**DOI:** 10.1007/s43545-021-00185-8

**Published:** 2021-07-06

**Authors:** Christopher P. Barrington-Leigh

**Affiliations:** Institute for Health and Social Policy and Bieler School of Environment, McGill University, Quebec, Canada

**Keywords:** Life satisfaction, Policy, Sustainable development, Happiness, Quality of life, Government finance

## Abstract

The growing maturity of the “science of happiness” raises the prospect of enabling government policy to be more accountable to the measurable subjective experience of the population. In its ideal form, the application of this science promises to inform decision makers about the likely distribution of life satisfaction resulting from any prospective policy, allowing for the selection of more optimal policy. Such “budgeting for wellbeing” invites three natural objections, beyond normative quibbles with the subjective objective: (1) non-incremental changes are unlikely in large bureaucracies, so a new accounting system for devising and costing government policies and budgets is too radical, (2) governments do not have an authoritative set of credible cost/benefit coefficients to use in analysis, and (3) long-run objectives, risks, and environmental considerations cannot be feasibly captured in quantitative projections of human subjective wellbeing. Three institutions are needed to address these challenges. I describe (a) an evolving collection of largely objective indicators for monitoring progress, with life satisfaction providing quantitative structure and overarching visibility to the system, (b) a publicly curated, evidence-based *Database of Happiness Coefficients*, and (c) independent public agencies that decide on a growing list of material constraints on the economy. Rather than overwhelmingly novel, these features have antecedents and analogues. Moreover, most civil service decision-making and projection-making apparatuses need not change. Also, there will be no less room nor less need for political debate and platforms. While shifting society to human-centred measures of progress may be radically transformative in the long run, it can be initiated smoothly and non-disruptively.

## Introduction and context

The effort to modernize metrics for progress and social-economic success is still often framed as moving “Beyond GDP”. Not only have statistical agencies, policy makers, and societies as a whole largely failed to realize the call to converge on a broader and more appropriate measure of human wellbeing, but ironically the “de-growth” movement, which associates GDP with environmental harm, has in once sense served as part of the same chorus as growth-centric development economists. That is, advocating for growth and advocating for de-growth both keep the focus on GDP. Instead, in order to truly move beyond GDP, the time has come and the tools are at hand to measure what matters more directly—both to gauge human wellbeing and to gauge impacts on the environment.

This paper answers key questions about a strong version of this vision, in which these measures are distinct and policy is accountable to them in distinct ways. It is premised on the existence of an indicator for human wellbeing that is meaningful enough to be a quantitative guide to decision-making in government. Life satisfaction—a transparent, compelling, comprehensive, and sensitive measure—appears to be such an indicator (e.g., Helliwell et al. 2012; OECD 2013; Office for National Statistics 2012; Randall et al. 2014; Tinkler 2015; Office of National Statistics 2011; Diener 1984).

Life satisfaction contradicts GDP in a number of ways when interpreted as a measure of progress. For instance, life satisfaction data have shown that a population might not become happier as it becomes richer, that an extra dollar of income going to a wealthy family has a measurably small impact on life satisfaction as compared with when it goes to a low-income family, and that the quality of relationships in a workplace matter more, on average, than does income. Life satisfaction data put a quantitative value on the importance of feelings like community trust and a sense of belonging, the psychological benefit of having a trustworthy government, and the emotional cost of being unemployed, which is much greater than the financial disruption alone. Life satisfaction data enable us to evaluate the relative benefits of addressing mental health problems as compared with other medical interventions, the lifelong non-monetary value of protecting children from adverse circumstances, the benefit of teaching social and emotional skills to people of all ages, and other investments in overall quality of life.

On the other hand, based on what is known about the determinants of life satisfaction, it seems feasible to imagine a society with high life satisfaction but which is running down the resources left for future generations. While a government decision maker can, if equipped with sufficient information, choose policies to nurture high life satisfaction in the near and medium term, there are limits to the scope of decisions that can be treated in such a wellbeing-driven framework. In particular, when future circumstances are outside the scope of past experience, or uncertainty is too high to carry out calculations and optimizations, or consequences from today run too far into the future, a wellbeing framework for policy making is likely to fail to provide sufficient confidence for decision-making.

A second danger looms. There is a tendency to create indices of progress or wellbeing which combine multiple, disparate outcomes with entirely arbitrary weights, leaving them indefensible upon scrutiny, often after attracting initial public and political attention (Barrington-Leigh and Escande 2018). Worse, such indices often conflate, i.e., add together, measures related to human experience with measures related to ecological limits. An example is the single (scalar) index created to track the highly influential U.N. Sustainable Development Goals (SDGs). It is a sum of 100 numbers, all treated as equally important, which cover the disparate ideas captured by the SDGs. De Neve and Sachs (2020) note that indices for SDG goals 12 (responsible consumption and production) and 13 (climate action) have a negative relationship with wellbeing. They conclude that “policy-makers may find pursuing [these] more difficult” as a result. Conflating measures of quality of life with those of ecological outcomes acts, like a focus on GDP, to buttress fears of a tension between progress and sustainability. Instead, these objectives must be rhetorically and conceptually separated in order to make sustainable development politically feasible.

The following complementary approaches address this challenge: (1) a system of constraints, particularly on material use and waste generation, acts to simplify decision-making about the far future, especially when it is characterized by high uncertainty; (2) within such constraints, government decisions can be informed by the best evidence on what makes for good current and medium-term future lives.

Several institutions, described below, will be necessary to realize this ideal. While the overall scenario of happiness-maximizing policy subject to physical limits represents a transformative change, most of the pieces are already in place, at least in an embryonic state. The sections below describe the following *existing* institutions:

1. The ongoing monitoring of subjective wellbeing by government statistical agencies;
2. Public databases of “happiness coefficients” which encapsulate knowledge about how much a particular change or difference in life circumstances is likely to improve or reduce an individual’s quality of life;
3. Government planning models of how events at one point in someone’s life affect their behavior, productivity, and need for government services later on in life;
4. Monitoring, accounting, and enforcement systems for implementing conservation constraints on the use of resources and emission of waste products.

With some further development of these institutions, they could together guide governments in making trade-offs between competing needs, while limiting long-run risks that may be said to define many of our sustainability threats.

This paper addresses the following two questions: Theoretically, how can evidence on human subjective wellbeing inform inter-temporal policy decision-making? What institutional innovation is needed to shift expectations and practice to realize that vision? The remaining structure is as follows: ‘What is “happiness”’ defines the terms and scope of the life satisfaction approach, while ‘Measuring wellbeing of society’ describes how to construct dashboards of wellbeing indicators that are accountable to evolving evidence and measurement availability. ‘The *Database of Happiness Coefficients*’ describes how this evidence may be curated transparently by civil society, academia, and government, resulting in the *Database of Happiness Coefficients*. ‘Investments over the life course’ describes how governments translate their real policy options into future outcomes that are covered by this database. The central ‘Sustainability is different from future happiness’ addresses the practical limitations of such planning for the future, necessitating a complementary approach for imposing sustainability constraints. ‘Cost-benefit budgeting’ describes how the preceding institutions are to be used in cost-benefit analysis for devising budgets or legislation, and how inequality and discounting of the future relate to the topic of this paper. ‘From here to there’ outlines how these institutions and new practices can come about over time, and ‘Conclusion’ concludes.

## What is “happiness”?

An international standard version of the *satisfaction with life* (SWL) question in English is:

The following question asks how satisfied you feel, on a scale from 0 to 10. Zero means you feel “not at all satisfied” and 10 means you feel “completely satisfied”. Overall, how satisfied are you with life as a whole these days? (OECD 2013)

The key to the life satisfaction approach in policy-making is the availability of a subjective measure of individuals’ overall wellbeing. Rather than build up and advocate for an index composed of a collection of one’s favored goals, life satisfaction data rely on individual respondents to report their overall experience, taking into account everything together in the right proportions. Then, statistical methods are used to unravel the importance of different contributions to a good life. Individually, respondents are not experts on how hypothetical changes to their lives would affect their future life satisfaction, but they are sole experts on how good their own lived circumstances feel. Collectively, many respondents living a variety of different circumstances can reveal which conditions foster the best lives.

In this paper *life evaluations*, *experienced wellbeing*, or simply *wellbeing*, all refer to respondents’ quantitative answers to the SWL question as a primary representative of the data informing policy makers about overall *quality of life*. It is common in the economics of happiness literature to gloss over a number of distinctions (OECD 2013) within the domain of *subjective* *wellbeing* (SWB), and sometimes even *happiness* is used informally to denote the evaluative variant (such as SWL) of SWB.

## Measuring wellbeing of society

Even with all the evidence on the psychological and economic validity of life satisfaction as a metric (e.g., Diener 1984; Sandvik et al. 1993; Saris et al. 1998; Frijters et al. 2020), using life satisfaction as a headline indicator for human progress is of course ultimately an ethical or philosophical choice. Nevertheless, it has strong rationale (e.g., Hall et al. 2011; Dolan et al. 2011; Barrington-Leigh 2016a, b; Barrington-Leigh and Escande 2018; Barrington-Leigh and Wollenberg 2019; Global Happiness Council 2018, 2019), even in the face of some objections (e.g., see Durand (2020), and other articles in the same special issue).

One way to think about SWL is as a headline indicator which may accompany a dashboard of other, more objective indicators (Barrington-Leigh and Escande 2018; Hall et al. 2011; Department of Finance Canada 2021). Reported in its raw form, SWL communicates the overall intent of an indicator system. Its subjective nature makes clear the primacy given to the lived experience of a target population.

Going a step further, SWL can be used to derive statistical evidence about the relative importance to wellbeing of each objective indicator. Although the process is not completely devoid of judgment, these statistical calculations, typically linear regressions, are open to scrutiny and subject to revision in light of ever-expanding evidence.

Thus, life satisfaction can provide accountability to the choice of an entire dashboard of indicators, reducing the need for the dashboard designers to impose their judgment about which policies, government departments, or domains of life constitute priorities for wellbeing.

Taking another logical step, a *scalar index* (i.e, one number summarizing a whole set of indicators) of wellbeing can be constructed from a dashboard of *objective* measures. The same statistical inference used to determine the importance of each objective indicator can be used to provide weights to aggregate those indicators into a single number.^1^ In this way one can avoid assuming arbitrarily that all components of an index are equally important (Barrington-Leigh and Escande 2018), as do numerous indices like the U.N. Human Development Index or attempts to rank SDG performance (Miola and Schiltz 2019; De Neve and Sachs 2020).

By extension, SWL data can also suggest which indicators to drop entirely from an index or dashboard. In this approach, if an indicator is included in a summary “wellbeing” dashboard or index, it should be because it is found to be important in causal statistical models of wellbeing, i.e., because it is useful in differentiating between those experiencing high quality of life and those experiencing low quality of life, overall. A hierarchy of indicators, or an overall index, organized around SWL thus has an intrinsic legitimacy in its conception and design. The value it embodies is clear, and the idea that policy should be targeted and accountable to improve such a measure is compelling. Its quantitative and transparent nature allows others both to understand and reproduce it.

In summary, SWL has a natural role both as a headline indicator in its raw measured form, and as an organising concept, based on transparent and falsifiable evidence, for a broader array of (more) objective indicators. Below in ‘Investments over the life course’ these objective indicators will represent intermediate policy objectives. The processes described above are not mechanical, because they involve empirical evidence and inference, and therefore interpretation and debate; however, they are accountable to this evidence base. This distinguishes them from other approaches involving expert judgment or democratic selection (see for example Barrington-Leigh and Escande 2018; Decancq and Lugo 2013, for reviews).

Being able to build an index out of objective measures has other advantages. Running representatively sampled surveys is always expensive, and life satisfaction data require particularly large sample sizes because life satisfaction varies in response to so many factors. Objective and community-level conditions tend in this sense to be less noisy and therefore less expensive to measure, so they can be measured more frequently or with more demographic or geographic detail than the SWL that is needed to estimate weights.

**Fig. 1:**
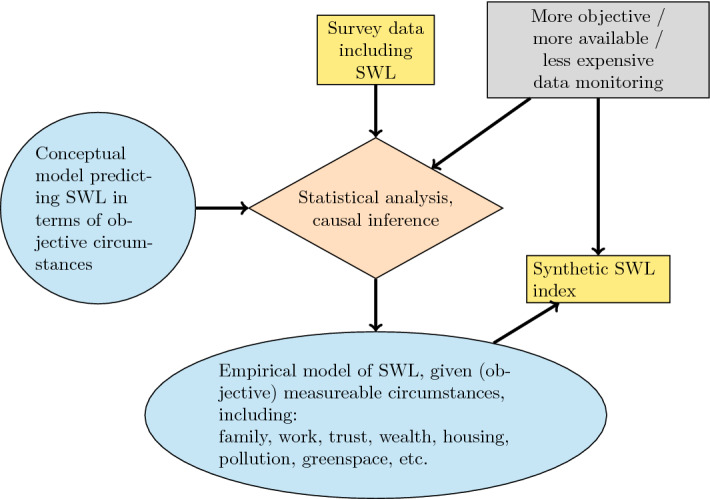
Measurement of life satisfaction (SWL) and generation of empirical weights for a wellbeing index

Figure 1 presents a conceptual depiction of how a “synthetic SWL” index (lower yellow box) can be constructed and published using the weights from accumulated knowledge about the determinants of SWL (lower blue oval). The key element is the survey measurement of actual life satisfaction reports (top yellow box), typically as part of a questionnaire which also assesses numerous other life conditions experienced by each respondent. The top right gray box represents these other life conditions, along with any other measurable life circumstances applying to an individual or her geographic region.

Many national statistical agencies are already measuring the life satisfaction of their populations. For example, Statistics Canada poses the question to more than 90,000 residents each year as part of comprehensive health and social surveys. At least seven countries include the question in national panel surveys; for instance the German Socio-Economic Panel study has tracked the life satisfaction of the same individuals over time, starting in 1984. The World Values Survey and the Gallup World Poll include overall life evaluation questions in their international surveys, which are in Gallup’s case annual and cover most countries.

## The *Database of Happiness Coefficients*

This wealth of data on people’s lives in a wide variety of circumstances within and among countries, including respondents undergoing a diversity of changes and life events, and subject to a variety of public policies and policy changes, has provided a rich base of knowledge about what makes life good.

This academic knowledge is in the form of a large body of published statistical analyses over several decades. Recently, it has begun to be collected into summary databases [60] (Frijters and Krekel 2021; Clark et al. 2019; Barrington-Leigh and Lemermeyer 2021) in a form that could help governments evaluate the impacts of prospective policy.

A “*Database of Happiness Coefficients* ” (coined by Happiness Research Institute 2020) contains the same weights, or “coefficients,” described above in the context of a dashboard of objective indicators. They tell us how someone, or some community, is likely to evaluate life given an objective description of their current life. In Fig. 1, the lower blue oval depicts the *Database of Happiness Coefficients* (*DoHC*).

Two key steps are needed to ensure that governments have access to a reputable database of these coefficients. First, internationally and possibly within each country or jurisdiction, it is incumbent on analysts to debate and distill knowledge about the relationship between policy-influenced variables and human experience, in an accountable and ongoing process. Frijters et al. (2020) describe a process for a transparent, public database of coefficients from the best available evidence, organized to encourage constant generation of improved evidence. They mention the IPCC process for aggregating scientific evidence on climate change coefficients as an example. They also provide a “preliminary list” of coefficient values, compiled by the UK-based What Works Centre for Wellbeing, as a demonstration that the science is mature enough for this approach to be viable.

The second key to the construction of a consensus model of the determinants of SWL is to increase the contribution of policy experiments and policy evaluations towards the evidence base. This means expanding the measurement and monitoring of SWL and its social supports. Exceptional effort should be made when a policy changes, or where a policy roll-out affects only a subset of the population or reaches different groups at different times. In this way more policy changes can be turned into policy experiments, typically through partnership with academia, by cleverly varying or randomizing who is initially impacted.

These elements can give rise to an accountable, open database growing in both confidence and scope, differentiated by country as needed or desired, which gives the best available estimates and confidence intervals for the effects of individual, social, and collective life circumstances on human life satisfaction. The curation task of the *DoHC* should, at least initially, be up to the scientific community and civil society, rather than government. Happily, precisely those appointed groups have already taken up the task.

## Investments over the life course

While a *DoHC* specializes in mapping current conditions to current wellbeing, policy focuses on affecting *future* outcomes. Coefficients in a *DoHC* can be used to predict, for example, the difference in satisfaction of employees in workplaces with different levels of trust, or the difference in satisfaction between people who had stable and safe childhood environments and those who did not. However, when it comes to policy, a government cannot instantly change citizens’ trust of coworkers, nor change the childhood experience of adults.

Instead, a government interested in happiness considers making *investments* now in order to affect the *future* outcome of trust or the *future* burden of carried traumas. How might developing a curriculum for conflict resolution in primary and secondary school, or making one mandatory for matriculation, affect trust levels in workplace environments some years into the future? How might additional spending on maternal and parent resources or child protection change the future impacts of childhood experiences? More generally, the question thus arises: how will intermediate outcomes evolve in the future, given the implementation of a particular policy rule, the provision of a particular public service, or the collective investment in a particular resource? Answering these questions more or less explicitly is already the task of each government department within their particular domain.

Those intermediate, future outcomes can, with a *DoHC*, be translated into future predicted life satisfaction. However, the dynamics of how investments in individuals, communities, and infrastructure will affect circumstances faced by individuals in the future—these are questions that must largely be answered independently of a *DoHC*. Indeed, these questions arise in the delivery of most policy, independent of any interest in subjective wellbeing. When government agencies justify specific expenditures on education, public health, rehabilitation, other social supports, or indeed on any civic infrastructure, it is based on a belief about how benefits will accrue in the future from those investments. Models of these dynamics are used all the time to choose between alternative uses of public resources, even if those models are sometimes quite simplistic.

As a result of the availability of longitudinal, linked, citizen-based data, such government models are becoming more sophisticated. In recent years, for instance, New Zealand has revamped a number of its social spending programs to use the best evidence on how social service investments in an individual lead to public savings over several decades. These calculations are focused primarily on achieving “a positive long-term financial impact for the social sector.” That is, investing in human capital now saves the government money in the future. However, as the government notes, this *investment approach*

“...also has non-financial benefit as people experience longer lives, lived in better health and independence, with greater educational achievement and with dignity. As a specific funding mechanism, ‘investment funding’ gives providers an incentive to focus on these long-term impacts and value them alongside immediate, short-term gains (p. 6, Minister of Health 2016).

An extensive *DoHC* is the ideal tool to evaluate those *future* benefits in human terms. In fact, both financial costs and benefits can be expressed in terms of their wellbeing implications through use of a *DoHC*, since government expenditures translate into increased taxes and livelihoods in predictable amounts, and these circumstances have implications for life evaluations.

Understanding investments over the life course of an individual requires coordination of efforts across multiple sectors of government. Importantly, expressing the benefit stream over time in overall quality of life terms, using the *DoHC*, puts into commensurable terms the cost effectiveness of spending across all government agencies. Thus, not only does the task require coordination and foster integrative policies, but it allows one department to value benefits of its services which normally accrue within the domain of another department—i.e., to properly value complementarities and synergies across offices, ministries, and jurisdictions. Ultimately, a common metric of performance can also facilitate wellbeing-based budgeting at the highest level.

To summarize, while the *DoHC* initially specializes in information about short-run relationships, government agency knowledge about medium-run returns to investment is what links current delivery and policy actions to future objective outcomes. Such investment may be in human capital, in communities, in infrastructure, and in the environment, and the future objective outcomes can be evaluated in human wellbeing terms through the *DoHC*.

## Sustainability is different from future happiness

Some environmental concerns are well addressed in the paradigm described above. The general investment logic is as follows. Governments use evidence-informed methods to decide to tax away some resources from today’s consumption in order to invest in, say, subsidized childcare or public housing. Such investments can be worthwhile on the basis of building better lives in the future in exchange for a small wellbeing cost today. The life satisfaction approach in principle allows for all the diverse costs and benefits to be added up and compared in a sensible way, informing a choice about the “right” amount to spend.

Such spending will naturally include many environmental investments. There is already a large set of studies within the subjective wellbeing literature that quantifies the impact of environmental goods on life satisfaction (Maddison et al. 2020). Therefore, many environmental exposure variables will naturally end up in the *DoHC*, and our understanding of how policy can affect those exposures in the future will inform certain environmental policies. For instance, exposure to noise, pollution, and green space appear to have an immediate, quantifiable, and sustained effect on life satisfaction (e.g., van Praag and Baarsma 2005; Levinson 2018; Ambrey and Fleming 2014). Reduction of exposure to lead, or ensuring the viability of a fishery, may be predicted to affect other life conditions, listed in the *DoHC*, over a generation. Thus, cumulative policy impacts on life satisfaction may be estimated based on those life conditions.

### When the calculus fails

However, some future outcomes are too complex to predict well. How might gradual topsoil erosion, land use change, groundwater depletion, or fossil fuel extraction be incorporated into a government decision-making framework? One untenable option is as follows. Abiding by some variant of the Brundtland et al. (1987) definition of sustainability, or by the logic of “weak sustainability” articulated by Solow (1991), we would ensure that, overall, the wellbeing of those in the future is at least as high as our own. We would project how current policy options would affect objective outcomes in the future, coupled with a *DoHC* to calculate the corresponding impacts on overall life quality. The goal would be to calculate the optimal level and kinds of consumption to maximize current well-being while ensuring that, taking into account the numerous other gifts we bequeath to our descendants, future generations would still have good lives overall.

That plan is a mirage. For long-run, unfamiliar, unpredictable, complex, and uncertain dynamics, these calculations are not feasible. In those cases, it is not possible to choose an optimum based on accumulated knowledge about returns to investment (‘Investments over the life course’) and the *DoHC*, because no consensus on sufficiently precise predictions will be possible. Thus, the wellbeing approach fails in these cases and, one might say, the domain of “sustainability” considerations begins.^2^ The rest of this section explains how using material constraints on human activities can address these sustainability considerations, without compromising the technical feasibility and conceptual clarity of a wellbeing approach for most policies.

While the approach oriented around quality of life and epitomized by the *DoHC* is in principle highly rationalized, preservation of complex systems—especially natural ones—need not be justified in terms of calculable impacts on human well-being.

For instance, reflecting on the contribution of academic economics to the question of how to manage greenhouse gases, it seems that two decades were squandered theorizing about the right discount rate and preference parameters which, if known, would point to a particular optimal combination of mitigating climate change versus adapting to it. Instead, had society been equipped already with norms and institutions for an alternative, precautionary approach, we could more easily have recognized that the optimal abatement question could not be precisely settled based on quantitative arguments about wellbeing.

### An approach to long-run risk

How, then, are we to incorporate a concern for long-run risk or conservation into a framework which privileges human wellbeing?

Above all, the answer is to be willing to separate them (Neumayer 1999; Stiglitz et al. 2009). There needs to be a second rationale, besides accountability to predicted changes in human wellbeing, that society accepts to justify limits. A sensible approach is to address long-run problems through physical constraints, rather than optimization of wellbeing, when these problems are too complex or risky to treat through a system of prediction and quantitative balancing of human outcomes.

For example, in the case of greenhouse gases, a plan to stop the expansion of emissions could have been put in place in the late 20th century while further studies sought better precision on the future risks.^3^ More generally, our extraction of material resources from the earth and our addition of material pollutants to natural reservoirs could be subject to controls, sometimes in the form of explicit limits, justified not by calculable future well-being but by a principle of conservation, or an aversion to rapid change in natural or complex systems.

The approach can be applied to governments at all levels with enforcement authority: a city may decide to limit the growth of its footprint; a regional government in charge of mining may put an annual quota on both extraction rates and surface damage; and a national government may limit use of each ocean resource. In each case, a quota could be designed at first to halt further expansion of the rate of material extraction or effluent release, in ignorance of an “optimal” rate. The quota may subsequently be decreased, year over year, or otherwise adjusted based on arguments about the stability of the resource, as ecological evidence is available.

Key features of a system of sustainability constraints are that (1) the constraints are related directly or indirectly to objective physical measures, not to human benefits or wellbeing, and (2) that the physical measures are particular to each resource or waste stream, rather than being aggregated into an overall measure of environmental status or damage.^4^

For the purposes of making a distinction between wellbeing-driven policies and those justified by conservation considerations, there is no need to proceed into the details of how physical limits are implemented. The feasibility of building a democratic consensus for a particular level of emissions or rate of emissions cuts, the feasibility of solving collective action problems across multiple governments, and the problem of mechanism design for implementing controls, all lie beyond the scope of this paper. The focus is instead on protecting a life satisfaction approach from being burdened by non-commensurable objectives that it cannot accommodate. It is for this reason that society must have a complementary principle by which to manage certain long-run risks. That principle relates to controlling change, especially in natural resources and systems, when future implications of current consumption are unclear.

Without a set of principles and practices for dealing with sustainability issues, the policy reorientation towards wellbeing, described in prior sections, would be impracticable. That is, any realignment of policy away from an implicit production-growth bias, towards something more accountable to human experience, will run into trouble if it does not recognize that this accountability has finite practical scope. The life satisfaction framework may be enormously integrative in comparison to preexisting approaches, but there must be a social expectation that some regulations will be justified on a different, precautionary basis.

The justification behind a physical limits framework is ultimately to slow the pace of change of natural support systems in the face of uncertainty. For questions that are in this sense sustainability issues, it is universally the case that the true social cost of an activity is unknown, or the natural dynamics are too fragile or complex to predict well, or the social dynamics are subtle or complex. In these cases, an important starting point is to control the pace of material effects on those systems.^5^

As mentioned above, this begs the question of how to implement such conservation-minded constraints. In the greenhouse gas case, for example, carbon neutrality has become a principled goal for firms, regions, and nations. Early action could have been to institute a steadily and predictably rising price of emissions, without initial knowledge of how high it should end up. A price instrument can adjust over time to meet a more quantity-based decarbonization rule, with the principle remaining one of sustainability rather than optimization of wellbeing. An established instance of that principle is again carbon neutrality, which does not relate to any particular level of human wellbeing; in this sense it is arbitrary. Acceptance of conservation constraints, and tolerance of uncertainty about the long-run costs to wellbeing, are key to this policy framing.

Within the space defined by such constraints, policy can continue to focus on maximizing human wellbeing using the life satisfaction approach. Thus, a system of constraints protects the depletion of natural stocks of many kinds, but within those constraints society is generally directed to improve human experience according to the best available knowledge.

**Fig. 2:**
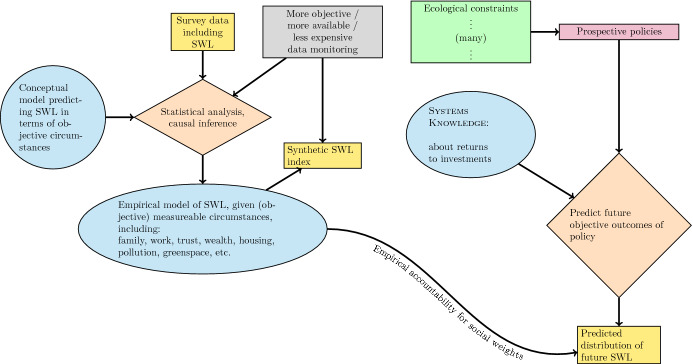
Components of a wellbeing-oriented policy-making process

Figure 2 depicts the combined institutions. The measurement and inferential processes which monitor the population and generate the *DoHC* are shown on the left. The green box represents sustainability constraints to policy, i.e., those necessitated by ignorance of certain long-run costs, and the “Systems Knowledge” oval represents the content of ‘Investments over the life course’, that is, the translation of prospective policies today into objective outcomes in the future. The *DoHC* in turn translates these into a population distribution of expected human experience, upon which preferences among policies can be based.

To reiterate the nature of the present proposal, let me point out that there is no description in this diagram of how to choose the stringency of conservation, such as the rate of convergence to zero for non-renewable extraction or pollution flows. The enormous literature on this subject remains relevant in the context of the green box in Fig. 2, and is not addressed here. Instead, my point is that there is a practical fallacy in casting all conservation considerations as components of wellbeing. This mistake can be avoided if public discourse admits a second principle for policy, using a conservation or precautionary rationale to justify the stabilization of ecological (or other) systems.

### Three possible critiques

#### A false dichotomy between wellbeing and sustainability?

Like most dichotomies, this one is not rigid. Governments already impose limits in the name of conservation, without embracing the dichotomy proposed here between wellbeing and sustainability. Material limits are most likely to be considered and introduced when there is a perceived risk to future human wellbeing. Later, when relevant natural and social science becomes sufficiently well understood that a calculus of future wellbeing can be applied, the material limit designed for ecological sustainability may be replaced by one fine-tuned for long-run wellbeing.

Conversely, every prospective policy comes with some risk, i.e., an imperfect prediction of its future consequences to human wellbeing. Predictions are, technically speaking, distributions of probabilities over different possible outcomes. For instance, a government model of human life course development may recognize some uncertainty in life expectancy of current generations and in future immigration flows; these possibilities will be reflected in a range of expected policy outcomes expressed in terms of wellbeing.

In part for this reason, there will always remain room for democratic will and political preference in policy, even in an environment where the population expects justification in terms of, and accountability to, a quality of life measure. The difference between this uncertainty in future wellbeing and that which motivates a physically denominated limit to conserve some resource is *in principle* only a matter of degree; however practically speaking two separate rationale—human wellbeing and principled conservation—are easier to understand and, I suggest, to institutionalize.

The dichotomy also has some internal coherence. Stabilizing natural systems and shifting to a reliance on sustainable resources may help to reduce uncertainty about the structure of life in future decades, thereby facilitating the kind of projections needed for a wellbeing approach to other policies. Conversely, focusing on an optimistic, quality-of-life-oriented discourse within the context of some material constraints should make the principled imposition of those constraints more palatable for all involved.

Lastly, in some contexts, a commitment to conservation principles is likely to buttress social cohesion and identity, and in turn life satisfaction. Indeed, an important support for life satisfaction is the degree to which people feel a connection to a meaningful social identity and a sense of cultural continuity (Chandler and Lalonde 1998). Another is the opportunity to act in support of others, which is a powerful promoter of individual wellbeing (Aknin et al. 2013). While the cultural benefits of embracing a principled conservation policy may be as difficult to calculate as the anthropocentric environmental benefits, they may be considerable. One might speculate that the promise of separating policy rationale about individual and collective happiness from stories about conservation may open the door to more narrative approaches, maybe akin to those which Indigenous peoples have used for millennia, for explaining the imposition of resource limiting rules. That is, allowing conservation constraints to be portrayed as part of a people’s identity rather than subject to arguments about wellbeing may have some immediate benefits for people’s wellbeing.

#### Unbounded costs to conservation?

Another possible critique of my argument is that the costs to wellbeing of an unnecessary or overly conservative constraint may be just as high as the potential damage of not imposing controls. There are two important premises which may make the physical limits approach compelling in the face of this concern.

The first relevant premise is one of the major insights from life satisfaction research. It is that the scope for improving, or indeed diminishing, life experience through *non-material* changes to society is enormous, while the scope for changing lives through *material* means is relatively limited (Barrington-Leigh 2016b). This may be counter-intuitive in the context of developing economies; nevertheless, the evidence spans all levels of development. Projections based on past development suggest that changes in GDP per capita and healthy life expectancy between now and 2050 are unlikely to change world average life satisfaction by even 1 point on the 11-point scale ( Barrington-Leigh and Galbraith 2019). By contrast, different feasible trajectories of a few non-material variables by 2050 account for a variation of nearly 3.5 points on the same scale, with the optimistic end leaving the average country as happy as today’s Belgium and Costa Rica. One interpretation is that the scope for improving lives may be surprisingly undiminished under the imposition of some material constraints.

The second proposition in defense of precautionary constraints is that on moderate time scales, innovation partly compensates for supply limitations. When material constraints are transparent and predictable, markets respond appropriately through innovation and substitution. The idea that such constraints can spur innovation so strongly as to be beneficial even in the short term (Porter and Van der Linde 1995) has support in a variety of contexts, although it will not apply universally. Nevertheless, the innovation bred by transparent constraints on a given material flow will always increase efficiency in the use or production of the constrained material, and will always mitigate the reduction in consumption benefit that would otherwise be experienced. We can be certain, for instance, that had oil become expensive 100 years ago, wind and solar power technology and electric transportation infrastructure would have advanced much earlier than it has. Policy should therefore focus on optimizing human wellbeing within a set of ecologically motivated constraints, rather than giving undue focus to opportunity lost to those constraints.

#### Sustainability problems not solved?

Another possible objection to the proposal of this section is the opposite of the previous one. It is that constraining resource extraction or pollution does not necessarily entail constraining it sufficiently. While true, this critique is more relevant to specific approaches to instituting consumption constraints, rather than to the general idea of imposing them.

Different environmental control instruments are appropriate in different situations. In instituting such protections, there are plenty of problems to do with free-riding across jurisdictions, intermingled with those to do with public will. However, these are likely either ameliorated or unaffected by implementing the ideas in this paper, which emphasizes separating a physical or ecological rationale for policy from one based on the science of wellbeing. If a public accepts a wellbeing-subject-to-limits approach, and if the institutions to enforce limits are in place, then updating limits in light of new ecological science, for instance, may be easier than debating the social costs and benefits of running down a natural stock.

### Precedents for physical limits

Fortunately, as with the institutions described in earlier sections, the institutions for limiting physical throughput are not entirely novel either in concept or in practice.

A number of resources are now capped at non-zero values. For instance, water extraction quantities, SO$_{2}$ emissions, fishery catches, forestry cut volumes, urban development perimeters, and CO$_{2}$ emissions are examples of material flows subject to caps, often allocated by auctions of tradeable quotas, or other material controls.

The idea has been around for even longer, but the proposal for widespread use of quotas to limit many principal material flows is due to Daly (1973). He recommended that quotas converge toward levels that abide by certain principles of sustainability for renewable and non-renewable resources (Daly 1990). In some cases these are practicable; in others, however, those levels suffer from uncertainty in natural or social sciences, just like insufficiently informed future wellbeing calculations. In both contexts, science will inform better targets over time.

Pigouvian taxes, i.e., taxes on environmental externalities, also have a long pedigree. In many situations the optimal instrument provides price certainty in the short run but is adjusted to meet physical constraint objectives in the long run. An example is the Western Climate Initiative’s carbon pricing approach for Quebec and California.^6^ In any case, the key relevant feature in a wellbeing policy framework is that there is an expectation that principled conservation criteria, not social costs, may be used as justification for limits.

In practice, international competition and political pressures will limit how stringent governments are willing to be in imposing controls. Nevertheless, expanding institutions and social acceptance for self-imposed limits expressed in physical and ecological terms, rather than those justified by projected human benefits, is an important complement to wellbeing-based policy making.

## Cost-benefit budgeting

While a *DoHC* will always be subject to further evidence and refinement, it can in principle be used to calculate (predict) the full distribution of predicted life satisfaction responses for a population or subpopulation. That is, the predicted outcome of a policy is not a single value (for instance, the average life satisfaction of the population), but rather a prediction of the entire population’s responses, as if every resident were asked the life satisfaction question at an appropriate future time, after implementation of the policy.

A government or society must choose what “moments” of this distribution it wishes to maximize. For instance, it could target the average (akin in ethical narrowness to pursuing a higher GDP, or average income), or the median, or any more complex aggregate, in which some extra emphasis is invariably given to the improvement of the lives of those at the bottom of the distribution. In addition, various inter-group differences in wellbeing outcomes will be politically important, just as they are now using less wholistic measures of wellbeing. Policy and preferences about distributional issues are thus no more nor less complicated than when choosing measures of income inequality. The discussion to follow continues to abstract from this issue by referring simply to life satisfaction as though there exists a clear preference on how to aggregate it across population and time.

In principle, under the framework of Fig. 2, any *legislation* may be tested for whether it is predicted to improve life satisfaction. Because the effect of extra taxation on life satisfaction can be estimated, any new government *expenditure* on services or investment can also be tested for whether it is predicted to improve life satisfaction. That is, the wellbeing cost of having less after-tax income may be added to the wellbeing benefit of the prospective new service. For investments it is possible that the answer might be different on the short term versus the long term. In any case, effects must be appropriately summed over time, so that the units of benefit-cost accounting become SWL over time, or some moment of the distribution function of SWL over time. This unit has recently been named WELLBY (Frijters et al. 2020; Frijters and Krekel 2021) or WALY (Happiness Research Institute 2020).

Some authors have suggested that this approach is unrealistic because the size of government budgets is set politically (Frijters et al. 2020; Layard and O’Donnell 2015), in which case a *cost effectiveness* version of the benefit-cost accounting becomes appropriate (Layard and O’Donnell 2015). In this approach, prospective policies can be ranked by a ratio of their anticipated effect on life satisfaction divided by their cost. The highest-ranked policies should be pursued, continuing until the budget is used up.

Layard and O’Donnell (2015) do not give any support for their premise that the size of government budgets cannot also be set with an eye to their effect on wellbeing, and that idea appears to be too conservative. Because of the complexities involved in the “Knowledge of Dynamics” component in Fig. 2 (‘Investments over the life course’), there will be no unique, mechanical answer to the question “How large should the budget be?”. Thus, plenty of room for political debate, heterogeneous policy regimes, and experimentation remains, even within a culture which expects the size of the budget to be justified in terms of wellbeing in principle.

Lastly, it is important to note that the idea of holding policy, and budgets, accountable to measured happiness is not novel (e.g., Layard 1980; Donovan et al. 2002; Layard 2006; Ng and Ho 2006; Cameron 2010; O’Donnell and Oswald 2015; Dolan and White 2007; Global Happiness Council 2018, 2019; Frijters et al. 2020). In the annual Global Happiness and Wellbeing Policy Reports, advice is being collated on best policies for happiness in education (Seligman and Adler 2019), healthcare systems (Peasgood et al. 2019) city-level policy making (Bin Bishr 2019), central government institutions (Durand and Exton 2019), and other domains. Being discipline-specific, these best practice guides integrate the kinds of knowledge and time frames characterizing both blue ovals in Fig. 2.

## From here to there

Superficially, a change towards a policy environment that is accountable to a human-centred measure of wellbeing, such as life satisfaction, may come across as intimidating to existing government analysts and policy makers. Indeed, considerable attention to capacity-building will be needed (Durand and Exton 2019) to make new analyses feasible. However, there is no need to conceive of a sudden nor threatening revolution. New Zealand has implemented a “Wellbeing Budget” which consists only of requiring federal departments to provide a structured evaluation of projected impacts for all budget submissions. The impacts are assessed across 12 prescribed domains of wellbeing and four kinds of capital which sustain wellbeing. These domains of “wellbeing” are not derived in an empirically accountable way from measurements of life satisfaction, but the initiative has a lot in common with how a life satisfaction approach would be unveiled in a budgeting process.

As mentioned in ‘The *Database of Happiness Coefficients*’, the *DoHC* always remains incomplete and subject to revision whenever new evidence can refine or extend the database. As governments become used to assessing outcomes in terms of subjective life evaluations, they will have extra incentive to take up the habit of engaging in experiments. New policies can be piloted on a limited population, in partnership with researchers and with careful monitoring of outcomes including life satisfaction surveys, or deployed sequentially in a way that facilitates causal inference. In this manner, experience can be pooled to support the breadth, confidence, and growth of the *DoHC*.

How might a government and public service get from a system with limited capacity for cost/benefit analysis to a policy regime that is quantitatively guided by human-centred outcomes, and simultaneously consistent with long-run commitments? I suggest three conceptual phases, described below. This account is meant to be illustrative and agnostic to a choice of political system.

### Short term: Evidence-based budgeting

The beginning of a transition sets in motion the shift in public expectations towards meaningful human-centred outcomes, and commits government down the conceptual and practical path of making policy, where feasible, accountable to the best evidence about human wellbeing. Once introduced, subjective reports are likely to retain a prominent position, since people’s overall experienced quality of life provides a compelling and empirically accountable principle for making public investments.

This first phase could therefore involve (1) rhetorical framing of a budget around **“evidence-based budgeting**” or a shift to evidence-based policy, along with the mention of overall life evaluations as an ultimate form of accountability for social outcomes; (2) ensuring that life satisfaction and associated key measures of trust, engagement, meaning, and time use are being measured sufficiently and regularly; (3) putting in place infrastructure to be able to **measure and monitor the outcomes** of policy changes and interventions and new allocations of resources; and (4) an improvement in capacity and standards for carrying out **quantitative projections for future evolution of existing objective goals** in each ministry or department.

The last item involves developing procedures for benefit-cost accounting and expanding the breadth of use of such approaches to include impacts shown to be important to life satisfaction but under-emphasized by existing practice. More significantly, however, it involves the implementation of investment models describing human, social, and physical capital to inform such accounting. This is the “dynamics” of ‘Investments over the life course’. How do investments in social supports at different points in an individual’s life play out over the life course? The same positive knowledge is needed for health, including mental health, and for other government expenditures. Normative preferences will remain relevant through discount rates, modeling approaches and assumptions, and simply through the selection of investments to consider. Nevertheless, public investments will be able to be evaluated, with increasing sophistication, according to the prospective (and retrospective) provision of benefits over time. Moreover, these projections should increasingly be made fully transparent.

### Medium term: The *DoHC*, monitoring, and policy experimentation

As the capacity-building and reframing of public discourse described above are consolidated, new evidence on human outcomes can be compiled. This entails (1) monitoring life satisfaction and related outcomes more intensively; (2) turning new resource allocations and regulation changes into opportunities for experimentation; (3) support for an independent, transparent, and public *DoHC*; and (4) increasing use of the *DoHC* to inform choice of objective outcomes to model and to measure. This means finding the low-hanging fruit where conventional productivity and market consumption approaches diverge the most from a more encompassing analysis, and where the costs to improving wellbeing are small (Durand and Exton 2019). In addition, (5) experimentation with the implementation of material constraints must be carried out as soon as possible in order to facilitate the remaining components of the transition.

While there are inevitably costs to building government capacity and infrastructure for new procedures, a premise behind this plan is that these costs would be vastly outweighed by the benefits of better policy. The low-hanging fruit is likely to be cases where synergies confer benefits that were hitherto ignored. For instance, with small changes, a policy program may be able to boost local social capital in addition to its primary objective. Such non-threatening early successes are likely key to building momentum and supportive leadership (Durand and Exton 2019).

### Long term: Accountability to life evaluations, and constraints against long-run risk

In the long run, projected outcomes are translated into changes in wellbeing by reference to the *DoHC*, and decisions and budget allocations both within departments and among ministries can be made and communicated in light of their future sequence of expected benefits to subjective wellbeing. As mentioned above, even the level of taxation can in principle be evaluated based on the costs to experienced wellbeing it imposes and the benefits to experienced wellbeing from that which it can fund.

However, questions of distribution will remain an important component of political preference and debate. The transformative aspects will be transparency of rationale and future expectations from a given policy, leaving them open to public analysis and informed debate, and the selection of, focus on, and justification by outcomes that are meaningful to people and supported by evidence on life satisfaction.

Also on the long run, a coordinated suite of material constraints at all levels of geography and government (‘Sustainability is different from future happiness’) can be implemented. The goal for many of these will be to halt the growth of material impacts on complex systems and to shrink those impacts over time, rather than waiting for knowledge of the optimal level of extraction or pollution based on human wellbeing. This means that the models, projections, and accountability based on the *DoHC* can remain tractable, without being overwhelmed, quantitatively or in terms of institutional capacity, by overly complex or uncertain projections, or overly long-term outcomes. It also allows room for principled policies, reflecting values and identity beyond quality of life, at least insofar as they relate to long-run values and conservation.

## Conclusion

This paper does not address the complexity of policy-making in hierarchical institutions, the pitfalls of alternative approaches, monitoring and enforcement costs for material constraints, the challenges raised by international trade and non-cooperation, or the additional complexities of distributional issues or temporal discounting. However, each of these aspects is already under consideration in the context of wellbeing-driven policy or being actively worked on (e.g., Happiness Research Institute 2020; Global Happiness Council 2019; Frijters and Krekel 2021) or will remain relatively unchanged by a transition to a wellbeing-led framework.

The intent here is, first, to convey the sense that the science and economics of happiness is mature enough to support a global re-orientation of policy-making; second, to fill in the missing piece of how such a world can approach sustainability questions that are not yet sufficiently amenable to the life satisfaction approach; and third, to explain the role of an institutional layer dealing with “medium term” dynamics between policy decisions and the known determinants of life satisfaction. Each of the necessary institutions already exists at least embryonically, allowing for an incrementalist transition to embracing a new, wellbeing-centred policy approach. The task of transforming governments towards accountability to more human-centred measures of wellbeing cannot take place as a sudden revolution, but as a mutually reinforcing evolution of public expectation and government practice.

Life satisfaction can act as an organizing concept for measuring human-centred outcomes and their distribution, and can provide empirical accountability both to the selection of a broader dashboard of objective indicators, and to important parts of the policy development and selection process. However, designing policy to optimize predicted human wellbeing is entirely insufficient to achieve sustainability, hence the complementary approach for long-term risk described in this paper. Indeed, in “moving beyond GDP,” it appears to be as important to properly situate ecological concerns as it is to choose a sensible measure of life quality. The common mistake of assigning extra, rather than less, meaning to GDP (Brauer et al. 2005) by targeting a decrease in economic value rather than in material effects, and the even more common mistake (e.g., Knight and Rosa 2011; Talberth et al. 2006; Bleys 2008) of anthropomorphizing the environment by trying to integrate its health into indices of the wellbeing of humans (Neumayer 1999), are both confusing and insufficient in strength or specificity of protection for ecological integrity.

Two ideological transformations are thus needed in public discourse. First, a reorientation of social and economic policy towards the subjective experience of humans and its evidence base, and second, an acceptance of ecological limits without an explicit justification in terms of human wellbeing, but which are instead denominated in ecological terms. Fundamental to my optimism that populations will embrace the second rationale is a belief that the first reorientation will reap large benefits to wellbeing through non-material domains of life, thereby coming to understand that ecological limits do not pose a strong threat to such wellbeing. However, an embrace of physical limits may also come about more directly, through gaining familiarity with carbon neutrality policies, for instance, even as support for them is driven significantly by fear of threats to humans.

Over time, the public will increasingly look to life satisfaction as a prominent, or headline, indicator of the state of society, and as a measure of the differences between subgroups in overall experience. Also, with access to the same independently curated *DoHC* on which the government relies, civil society will be able to evaluate the government’s rationalization of its policies using a common language and a sensible objective. Whether this revised objective leads to subtle or transformative changes over the long run remains to be seen; I would gamble on transformative.

For those concerned with the decoupling of growth and environmental impacts as an impossible challenge, any economic growth under the “beyond GDP” institutions described here would by construction under constant or decreasing caps be entirely decoupled from material flows. Happily, under a system with an explicit objective to improve life satisfaction, there may be very little public attention on GDP growth or contraction, because data on more compelling and relevant measures would be at hand.

While providing a new level of accountability to policy, this framework accommodates plenty of breadth for public debate and for creativity and diversity in policies and political platforms. This is due to two factors. The first is the complexity of ideas about how economic and social outcomes of policy will evolve over time, i.e., the dynamics of investment into individuals as well as infrastructure, which in principle may encompass much of the social sciences. The second is the existence of normative debates about how to deal with distributional issues, i.e., inequality, in wellbeing or in other intermediate outcomes.

I conclude that the key institutions described in this paper already have real-life precedents. The practical successes and lessons from existing sub-national implementations of material caps are valuable in designing a more comprehensive system of such constraints, but ultimately any transition rests on new institutions becoming accepted and expected by the public. Due to the convergence of a maturation of happiness research, wide concern about global climate change, and a global pandemic requiring reflection about institutional norms and about core trade-offs in the drivers of wellbeing, the time may be at hand. A sensible and intuitive approach is to enforce material constraints embodying ecological precaution and to optimize the quality of human lives within those constraints. With the framework described here, both parts of this combined task can be carried out quantitatively and with increasing transparency.

## Footnotes

1 Or a distribution over a single number representing wellbeing. Ultimately, the life satisfaction approach predicts the population distribution resulting from a given policy. It is then up to the usual normative rationale to decide whether one distribution is favorable over another, likely taking into account disparities between specific sub-groups of interest in the population.

2 I am not proposing to define sustainability, and I refer to conservation, sustainability, and precautionary principle loosely and interchangeably in this section. The important distinction is between wellbeing optimization and another, complementary, principled rationale, which I suggest should be conservationist.

3 If the greenhouse gas example sounds somewhat far-fetched, it is likely because there is an additional challenge in the case of global public goods. The resulting collective action problem confounds the present discussion on government policy making because national governments cannot be relied on to enforce global decisions. In fact, the lack of international enforcement and coordination will also complicate placing limits on extracting raw materials which are traded or which are embedded in traded goods.

4 Thus, it is useful to control the amount of bauxite mined each year, but not to limit the total amount of economic activity, nor to trade off bauxite mining for, say, groundwater conservation by combining the two in an integrated ecological index of some kind and imposing a limit in terms of that index.

5 A similar approach may be applied to other, non-material changes for which there is enormous uncertainty in wellbeing implications. In some sense, this is why some social and political structures are embedded into constitutions—to restrict rapid change. Physically limited resource flows could be thought of as constitutional limits circumscribing the conduct of welfare-improving activities.

6 The greenhouse gas case has shown us, again, that social costs are not only a subject of endless calculation; they can also be easily and radically manipulated, such as when the U.S. Environmental Protection Agency’s value dropped by one to two orders of magnitude in 2017 under a new administration. A principled approach committing to carbon reduction in physical terms is subject to political and public debate and leadership, but less subject to complex calculations like the EPA’s. See https://www.economist.com/united-states/2017/11/16/the-epa-is-rewriting-the-most-important-number-in-climate-economics.
